# UQCRFS1 serves as a prognostic biomarker and promotes the progression of ovarian cancer

**DOI:** 10.1038/s41598-023-35572-z

**Published:** 2023-05-23

**Authors:** Qiran Sun, Jiaqi Li, Hao Dong, Jiao Zhan, Xiaoming Xiong, Jiashan Ding, Yuan Li, Linsheng He, Jing Wang

**Affiliations:** 1 Medical College, Nanchang University, Nanchang, Jiangxi China; 2 Department of Obstetrics and Gynecology, The First Affiliated Hospital of Nanchang University, Nanchang, Jiangxi China; 3 Department of Obstetrics and Gynecology, First People’s Hospital of Foshan, Foshan, Guangdong China; 4 Department of Nursing, The First Affiliated Hospital of Nanchang University, Nanchang, Jiangxi China

**Keywords:** Biomarkers, Diseases, Cancer

## Abstract

UQCRFS1 has been reported to be highly expressed in gastric and breast cancer, but the mechanism remains unclear. The prognosis and biological functions of UQCRFS1 in ovarian cancer (OC) have not been evaluated. The expression of UQCRFS1 in EOC was detected by GEPIA and HPA websites, and the prognosis value was investigated by Kaplan–Meier analysis. Then the correlation between the UQCRFS1 gene and tumor-related signature were analyzed by Spearman correlation analysis and rank sum test. Subsequently, the expression of the UQCRFS1 gene in four ovarian cancer cell lines was detected. A2780 and OVCAR8 with the highest expression of UQCRFS1 were selected in the following biological experiments. Cell proliferation was detected by CCK8 assay, cell cycle and apoptosis were determined by flow cytometry, reactive oxygen species (ROS) production was detected by DCFH-DA, DNA damage gene mRNA expression was analyzed by RT-PCR, and AKT/mTOR pathway protein expression were also examined by western blot after siRNA transfection. We found that UQCRFS1 was high-expression in EOC and associated with poor prognosis. Spearman correlation analysis revealed that the high expression of UQCRFS1 is associated with the cell cycle, apoptosis, oxidative phosphorylation, and DNA damage. Further studies found that knockdown of UQCRFS1 cells reduced cell proliferation, cell cycle arrest at the G1 phase, increased proportion of apoptosis, ROS production, and expression of DNA damage genes, inhibited ATK/mTOR pathway. The study suggested that UQCRFS1 may be a candidated target for diagnosis and treatments in OC.

## Introduction

Ovarian cancer (OC) is one of the deadliest gynaecological malignancies and the leading cause of cancer-related death in women worldwide^1^. Cytoreductive surgery and chemotherapeutic drugs are the standard treatment for OC. Despite efforts being made in diagnostic methods and therapeutic strategies, the overall prognosis of OC patients remains pessimistic^2^. Hence, finding disease-related biomarkers and developing new therapeutic strategies for OC patients is urgently necessary.

Due to the role of mitochondria in carcinogenesis, they have received increasing attention in cancer research in recent years^3^. The mitochondrial electron respiratory chain consists of four enzyme complexes (I, II, III and IV), of which the stability of the protein subunits is essential to maintain mitochondrial function. The mitochondrial electron transport chain is often affected in carcinogenesis^4^. Excess reactive oxygen species are produced when the electron transport chain suffers damage, which is also an important factor in cellular damage^5^.

Here, we focus on the biological functions of the ubiquitin–cytochrome C reductase Rieske iron-sulphur polypeptide 1(UQCRFS1), an essential subunit of mitochondrial respiratory complex III in OC^6^. Deleting the gene encoding UQCRFS1 causes mitochondrial complex III deficiency, cardiomyopathy and alopecia totalis^7^. Preliminary evidence has shown that UQCRFS1 is high-expressed in both human gastric^8^ and breast adenocarcinoma^9^, and it has been identified as a prognostic marker for melanoma^10^. Moreover, UQCRFS1 knockdown also decreased mitochondrial membrane potential and weaker invasion ability in breast tumour cell lines. Few studies have reported the expression pattern and possible clinical significance of UQCRFS1 in OC except one which suggested UQCRFS1 was amplified as a homologous staining region (HSR) in OC cell lines and ascites samples^11^.

Consequently, the purposes of the current study were to determine the expression pattern and prognostic value of UQCRFS1 in ovarian cancer and explore the possible mechanisms affecting the development of ovarian cancer. We found that UQCRFS1 was high-expressed and further discovered that high expression of UQCRFS1 was associated with poor prognosis in OC, which may be attributed to cell cycle distribution, increased apoptosis, DNA damage gene expression as well as reactive oxygen species (ROS) production.

## Materials and methods

### Experimental tissue

The ovarian cancer tissues involved in the experiment were obtained from the First Affiliated Hospital of Nanchang University. This study was reviewed and approved by the Ethics Committee of the First Affiliated Hospital of Nanchang University (2022)CDYFYYLK (11-035), which confirmed that all studies were conducted in accordance with relevant guidelines/regulations, and informed consent was obtained from all participants and/or their legal guardians. Studies involving human study participants were conducted in strict accordance with the Declaration of Helsinki.

### Western blotting

Proteins were extracted from normal and ovarian cancer tissues. Tissues were dissolved in RIPA buffer (Beyotime, Shanghai, China) for 30 min with a mixture of protease and phosphatase inhibitors, and cell lysates were collected after centrifuging at 12,000×*g*, 4 °C for 10 min. The supernatants were used for western blotting. The protein concentration was determined using the BCA protein assay kit (Beyotime, Shanghai, China). Equal amounts of protein samples were thermally denatured at 100 °C for 10 min and then subjected to microgel electrophoresis using 12% sodium dodecyl sulphate polyacrylamide gel electrophoresis (SDS-PAGE). The separated proteins were transferred to polyvinylidene difluoride (PVDF) membranes by electroblotting at 200 V for 1 h. The membranes were then blocked with 5% non-fat dried milk powder for 2 h at room temperature, then incubated with primary antibodies against UQCRFS1 (1:5000, Abcam, Cambridge, USA), CDK2 (1:1000, Abcam, Cambridge, USA), CDK4 (1:1000, Abcam, Cambridge, USA), CyclinD1 (1:1000, Abcam, Cambridge, USA), pro-caspase3 (1:1000, Cell Signaling Technology, Danvers, USA), pro-caspase9 (1:1000, Cell Signaling Technology, Danvers, USA), Bcl-2 (1:1000, Cell Signaling Technology, Danvers, USA), Cyto-c (1:2000, Cell Signaling Technology, Danvers, USA), AKT (1:1000, Immunoway, Suzhou, China), phospho-AKT (1:1000, Immunoway, Suzhou, China), mTOR (1:1000, Immunoway, Suzhou, China), or phospho-mTOR (1:1000, Immunoway, Suzhou, China) The next day the membrane was washed three times with Tris-buffered saline with Tween 20 (TBST), then incubated at room temperature for 1 h with a secondary antibody (1:5000). Finally, proteins were visualized using an enhanced chemiluminescence detection kit. Statistical analysis was performed, and density values were assessed using Image J software ((National Institutes of Health, Bethesda, MD, USA). GAPDH (Proteintech, Wuhan, China) was used as the loading control.

### Real-time PCR quantification

Total RNA was extracted from OC tissues and cells using Trizol (Invitrogen, Carlsbad, USA) according to the instructions. RNA concentration was measured using a NanoDrop (NanoDrop Technologies, Wilmington, DE, USA), quantified to one thousand micrograms, and reverse transcription was performed using HiScript II Q RT SuperMix(Vazyme, Jiangsu, China). SYBR qPCR Master Mix(Vazyme, Jiangsu, China) and CFX Connect™ Real-Time PCR Detection System (Applied Biosystems, Hercules, CA, USA) were used to perform RT-PCR. GAPDH was used as the internal control. The primer sequences are listed in Supplementary Table S1.

### Immunohistochemistry

The expression levels of UQCRFS1 protein in 35 paraffin-embedded EOC tissues and in 10 normal para-ovarian tissues were analysed by immunohistochemistry (IHC). After dewaxing, rehydrating, and incubating the sections in sodium citrate buffer (pH 6.0), antigens were retrieved from the sections by heating in an oven. To inhibit endogenous peroxidase activity, they were blocked for half an hour in 3% hydrogen peroxide, then washed three times in PBS and incubated with primary antibody (rabbit anti-UQCRFS1, 1:200 dilution) overnight at 4 °C, followed by incubation with secondary antibody (anti-rabbit IgG, TransGen Biotech, Beijing, China) for 2 h at room temperature. After colour development with 3,3-diaminobenzidine (DAB), sections were counterstained with haematoxylin, dehydrated, fixed and sealed with a coverslip.

### Cell cultrue

The human EOC cell lines A2780 and OVCAR-8 were obtained from the cell bank at FuHeng BioLogy (Shanghai, China). All cells were authenticated by short tandem repeat (STR) profiling after receipt and were propagated for less than 6 months after resuscitation. A2780 cells were cultured in DMEM (Solarbio, Beijing, China) with 10% FBS (Every Green, Zhejiang, China), and OVCAR8 cells were cultured in RPMI 1640 (Solarbio, Beijing, China) medium with 10% FBS. These cell lines were maintained in a humidified chamber containing 5% CO_2_ at 37 °C.

### Cell counting kit-8(CCK8) assay

The proliferation of A2780 and OVCAR8 cells was evaluated using CCK8(Absin, Shanghai, China) according to the instructions. Briefly, cells were resuspended and counted, then seeded at 1 × 103/well in a 96-well plate. The medium was aspirated after 12 h, then 10 μL of CCK8 solution was added to the new medium. After incubating for 2 h at 37 °C in a 5% CO_2_ atmosphere, the absorbance was measured at a wavelength of 450 nm, and the time was recorded as 0 h. Readings were repeated on days 1, 2 3 and 4.

### Cell cycle analysis

Cells were collected by centrifugation at 800×*g* for 5 min, fixed with 1 mL of 70% ethanol in PBS at − 20 °C for 2 h, collected again by centrifugation, and washed an-other three times with phosphate-buffered saline (PBS). Cells were then treated with 20 μL of RNase A in a 37 °C water bath for 30 min, centrifuged at 1000×*g* for 10 min, then resuspended in 400 μL propidium iodide (PI, BestBio, Shanghai, China). After incubating for 30 min, flow cytometric analysis was performed using a flow cytometer (Beckman Coulter, Brea, CA, USA).

### Annexin V-PI assay of apoptosis

Cells were harvested by trypsinisation without EDTA, washed twice with cold PBS, centrifuged at 500×*g* for 5 min at 4 °C, resuspended in 100 μL of prechilled 1 × binding buffer, then washed with 5 μL Annexin V-FITC and 5 μL PI stain (TransGen Biotech, Beijing, China) in binding buffer and incubated for 20 min at room temperature in the dark. Then, samples were diluted with 400 μL of 1 × binding buffer and analysed by flow cytometry (Beckman Coulter, Brea, CA, USA). The proportion of apoptotic cells was measured by flow cytometry at an excitation wavelength of 480 nm.

### ROS production analysis

The reactive oxygen species (ROS) assay kit (KeyGen Biotech, Nanjing, China) uses the fluorescent probe DCFH-DA for ROS detection. DCFH-DA has no fluorescence and can be hydrolysed into DCFH by intracellular esterase, and intracellular ROS oxidize non-fluorescent DCFH to generate fluorescent DCF. Detection of DCF reflects the level of ROS in the cell. The medium was removed and the cells were washed with PBS, harvested by trypsinisation, then the cells were incubated with 10 μmol/L DCHF-DA for 20 min at 37 °C in the dark. After incubation the cells were washed twice with warm PBS and examined by flow cytometry (Beckman Coulter, Brea, CA, USA).

### Statistical analysis

All experiments were performed independently in three replicates. Data were analysed using SPSS and are presented as mean ± S.E.M. Comparisons between two groups were performed with Student’s t-test. Independent samples from different groups that did not fit the normal distribution were analysed using the Wilcoxon nonparametric test. Kaplan–Meier curves were compared using the log-rank test. Clinical data (age, FIGO stage, etc.) were used to adjust for the effect of UQCRFS1 transcription level on overall survival (OS) in OC patients by multivariate Cox analysis.

## Results

### UQCRFS1 is overexpression in OC and indicates poor prognosis

**Figure 1:**
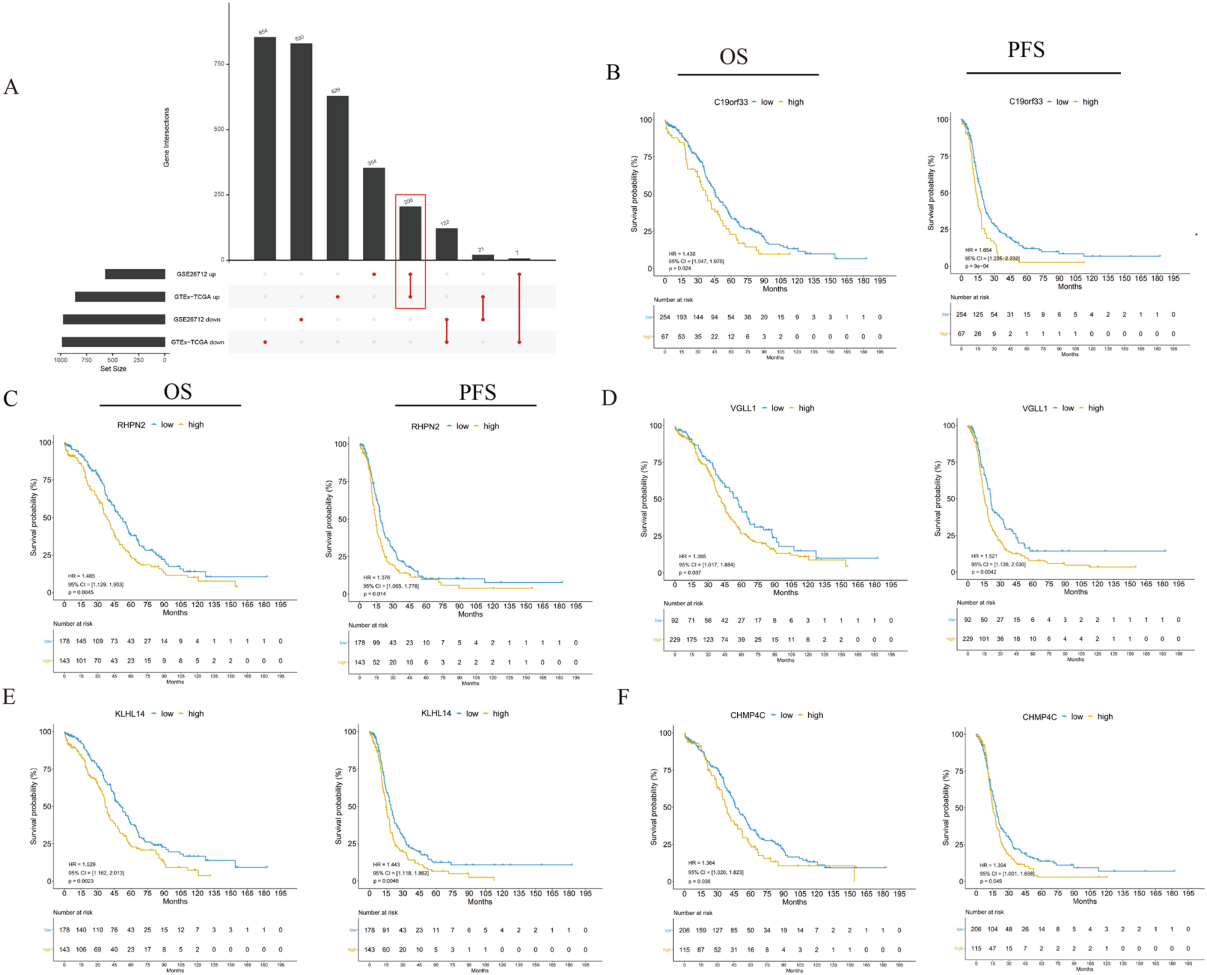
Identification of prognostic biomarkers in patients with OC. (**A**) The differentially expressed genes in ovarian cancer in TCGA and GSE26712 datasets were analyzed by limma package, of which 206 (DEGs) were highly expressed. 206 differentially expressed genes (DEGs) in ovarian cancer were screened from TCGA, GTEx and GEO databases. (**B**–**F**) Kaplan–Meier curves of overall survival (OS) and progression-free survival (PFS) of the 6 DEGs in patients with OC.

To identify differentially-expressed genes (DEGs), we retrieved the gene expression profiles from OC and normal ovarian tissues in TCGA, GTEx, and GEO. The GEO and GTEx databases were merged and named GSE26712. We identified a total of 206 DEGs using the ‘limma’ package (Fig. 1A). Then, we evaluated the prognostic value of the DEGs by the univariate Cox model, which revealed six DEGs which had an impact on the rates of both overall survival (OS) and progression-free survival (PFS) of OC patients, as potential prognostic biomarkers, namely C19orf33, UQCRFS1, VGLL1, KLHL14, RHPN2, CHMP4C. (Fig. 1B–F) (Supplementary Table S2). Subsequently, we performed multivariate Cox analysis to investigate the independent prognostic value of six DEGs (Supplementary Table S3). Among them, UQCRFS1, a critical subunit on the mitochondrial respiratory chain complex, was only reported in one case as amplified as homologous staining region (HSR) in OC cell lines and ascites samples. We selected UQCRFS1 as a prognostic biomarker for further investigation.

**Figure 2:**
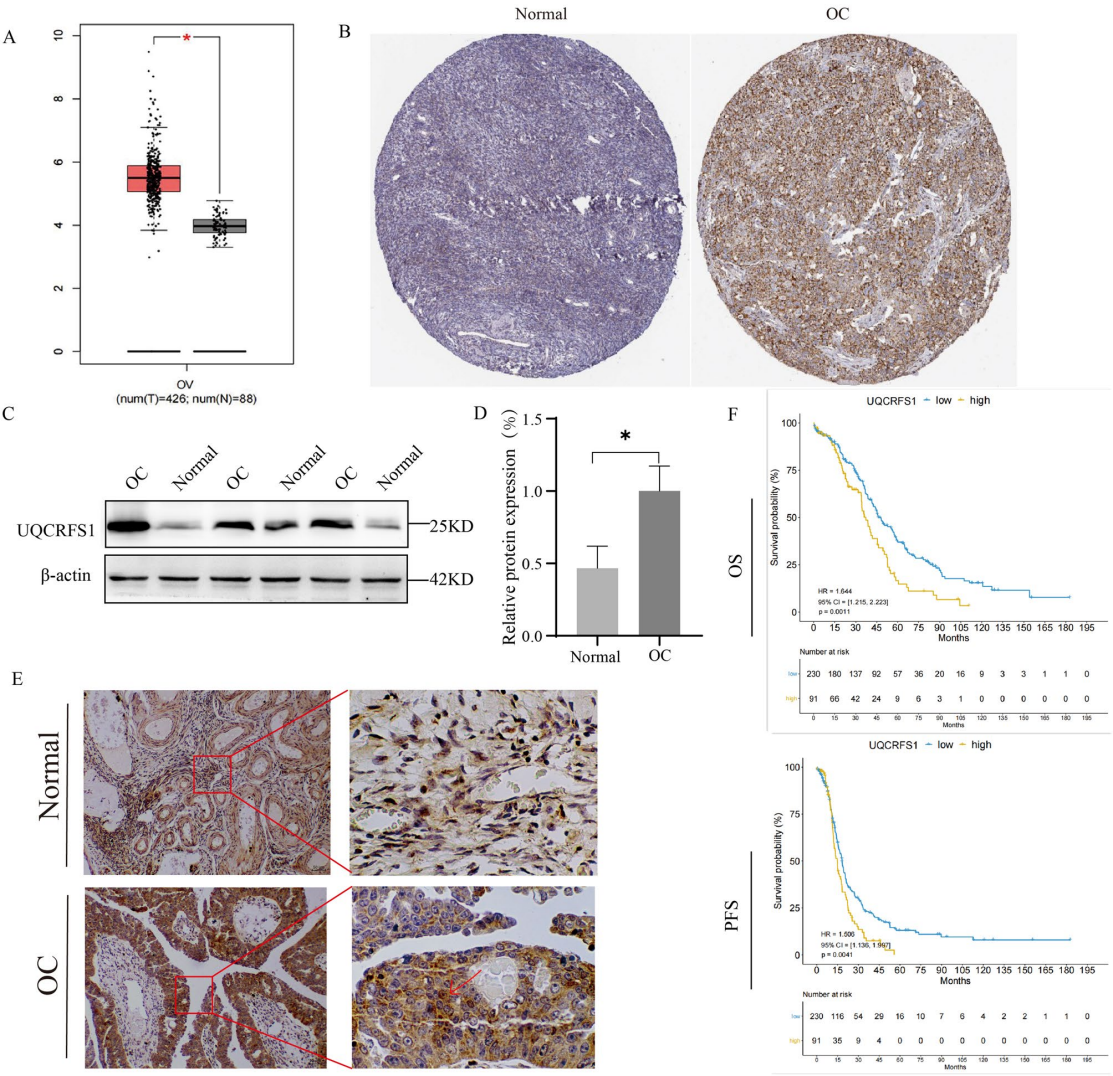
High expression of UQCRFS1 correlates with poor disease prognosis in OC. (**A**) Relative level of UQCRFS1 mRNA in ovarian cancer tissues (n = 426) compared with adjacent normal tissues (n = 88) from Gene Expression Profiling Interactive Analysis (GEPIA) online website. (**B**) Summary and representative images of UQCRFS1 immunohistochemistry staining intensities in normal ovarian and OC, based on Human Protein Atlas database (HPA). (**C**, **D**) UQCRFS1 expression evaluation by western blot analysis in normal ovarian tissue and OC (n = 6). (**E**) Representative IHC images of UQCRFS1 in OC and normal ovarian tissues (Normal = 10, Cancer = 30, Scale bar, 50um). (**F**) Kaplan–Meier curves of overall survival (OS) and progression-free survival (PFS) in ovarian cancer stratified by UQCRFS1 expression. In the bargraphs, the data are shown as the mean ± SD (*P < 0.05).

We evaluated UQCRFS1 gene expression using the Gene Expression Profiling Interactive Analysis (GEPIA: http://gepia2.cancer-pku.cn/) and Human Protein Atlas (HPA: https://www.proteinatlas.org/) databases. UQCRFS1 gene expression was upregulation in OC tissues compared to normal tissues (Fig. 2A,B). Western blot and immunohistochemical staining (IHC) analyses were applied in tumor and normal ovarian tissues to verify this observation. The result consistent with database. (Fig. 2C–E). Moreover, UQCRFS1 high-expression correlated with meaningfully poorer prognosis as revealed by Kaplan Meier-plotter analysis (Fig. 2F).

High expression of UQCRFS1 indicates a poor ovarian cancer prognosis. We hypothesized that UQCRFS1 is related to the occurrence and development of ovarian cancer.

### High expression of UQCRFS1 correlates with cell cycle, apoptosis, oxidative phosphorylation, and DNA damage

**Figure 3:**
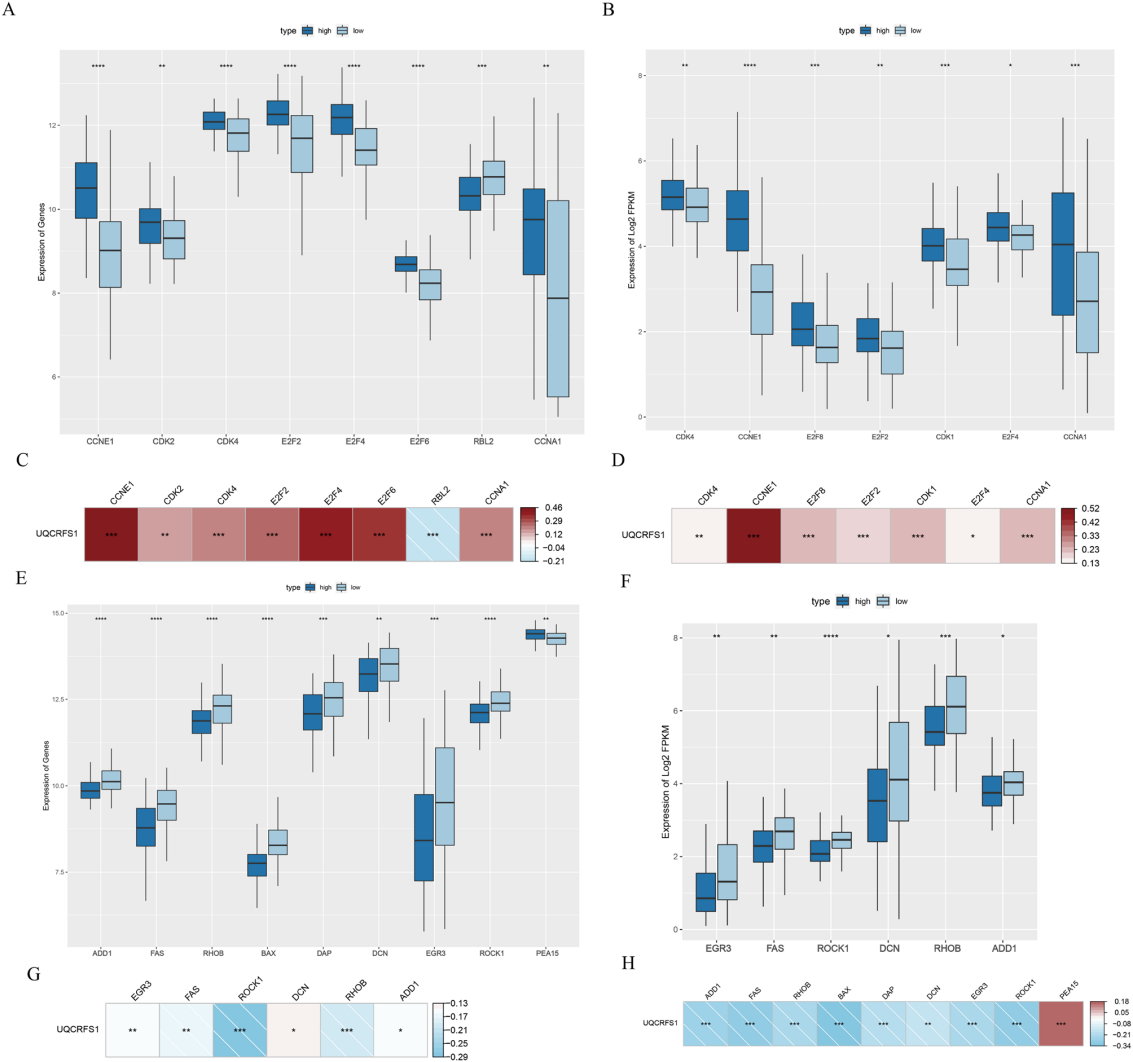
High expression of UQCRFS1 correlates with cell cycle and apoptosis. (**A**, **B**) The cell cycle genesets expression difference between UQCRFS1 high and low expression. (**C**, **D**) Correlation between cell cycle signature and UQCRFS1. (**E**, **F**) The apoptosis genesets expression difference between UQCRFS1 high and low expression. (**G**, **H**) Correlation between apoptosis signature and UQCRFS1.

**Figure 4:**
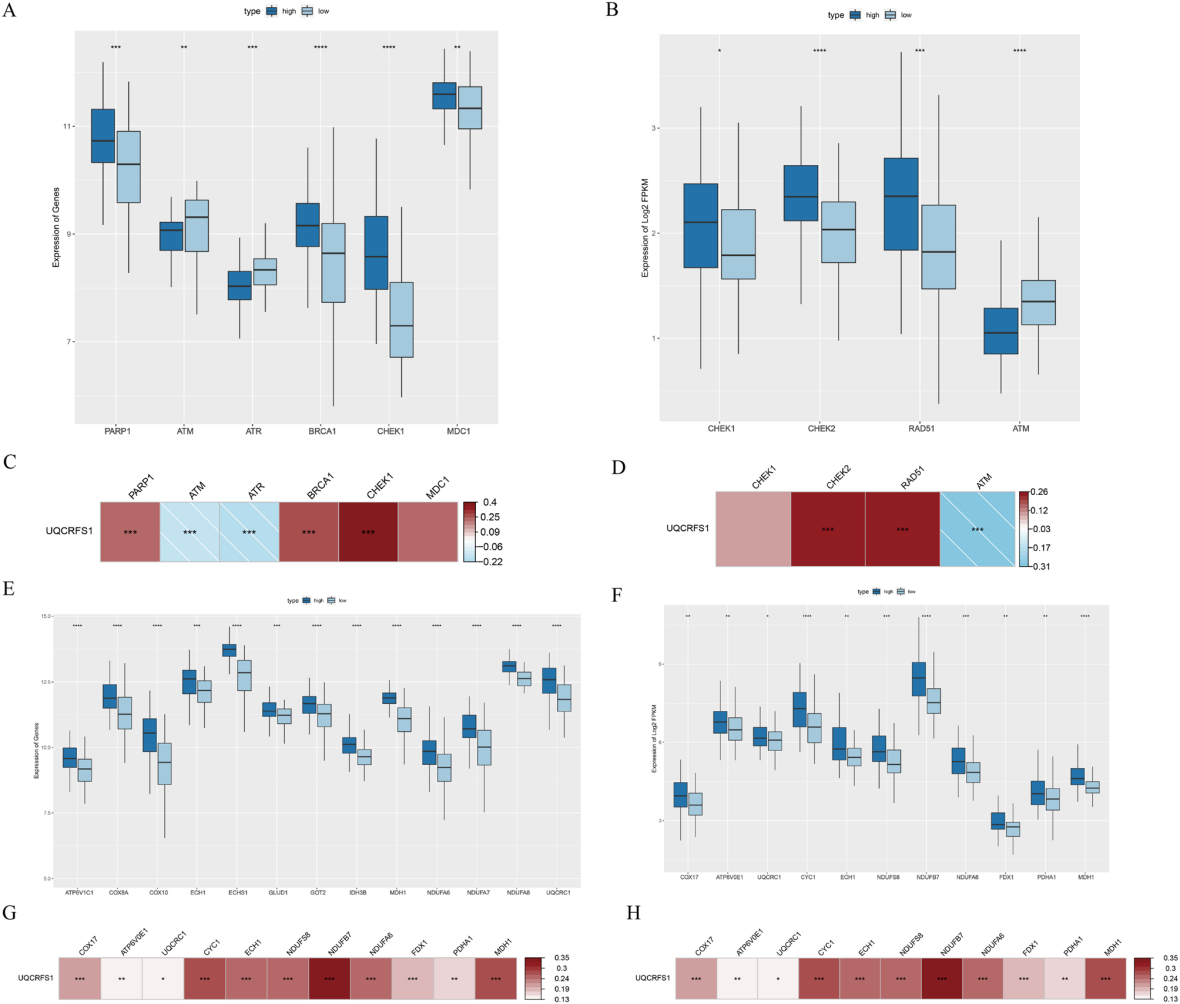
High expression of UQCRFS1 correlates with oxidative phosphorylation and DNA damage. (**A**, **B**) The DNA damage genesets expression difference between UQCRFS1 high and low expression. (**C**, **D**) Correlation between DNA damage signature and UQCRFS1. (**E**, **F**) The oxidative phosphorylation genesets expression difference between UQCRFS1 high and low expression. (**G**, **H**) Correlation between oxidative phosphorylation signature and UQCRFS1.

To gain insight into UQCRFS1, We downloaded tumour-related datasets from the c-BioPortal (http://www.cbioportal.org/) website, including cell cycle, apoptosis, oxidative phosphorylation (OXPHOS), DNA damage, and correlation analysis was examined with UQCRFS1 gene by rank sum test and spearman correlation analysis method. The result reveals that UQCRFS1 mRNA expression positively correlated with the cell cycle (Fig. 3C,D), OXPHOS (Fig. 4G,H), and negatively correlated with apoptosis (Fig. 3G,H), DNA damage (Fig. 4C,D). The UQCRFS1 upregulation group had a higher expression level of cell cycle genes (CDK2, CDK4, CCNE1) than the downregulation group (Fig. 3A,B), while apoptosis (ADD1, BAX, FAS) (Fig. 3E,F), and DNA damage (ATM, ATR) (Fig. 4A,B) genes were low-expression. Therefore, UQCRFS1 high-expression might play a tumour-promoting role. Moreover, the expression of OXPHOS genes was elevated in the high-expression group (Fig. 4E,F) and positively correlated with UQCRFS1 high-expression. It confirms that UQCRFS1 plays a critical role in maintaining the OXPHOS function.

The present findings shed light on the association of UQCRFS1 with various tumour features in OC and may serve as a potential biomarker.

### High expression of UQCRFS1 promotes proliferation of OC cells

**Figure 5:**
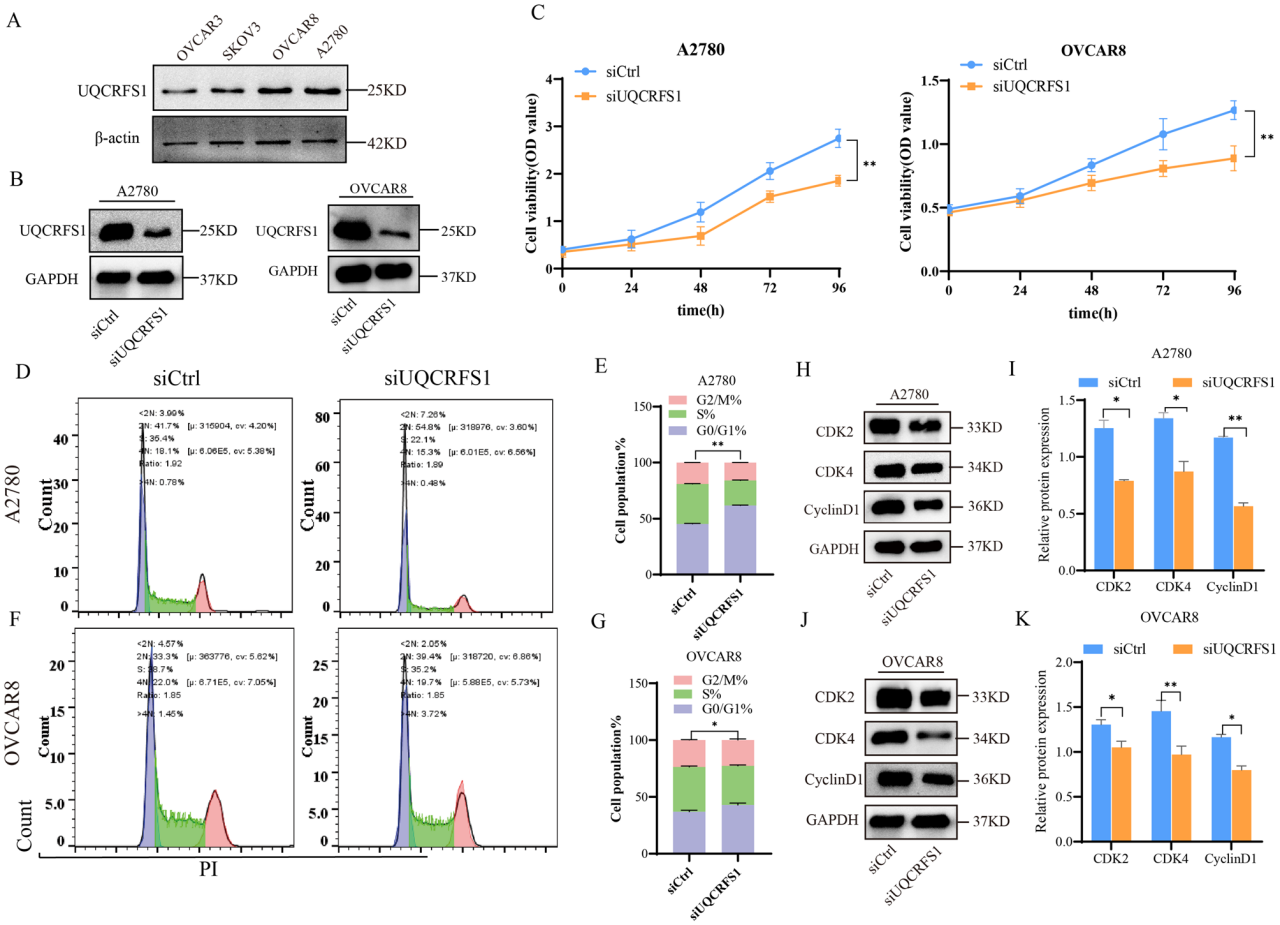
High expression of UQCRFS1 promotes proliferation of OC cells. (**A**) UQCRFS1 expression was determined in four OC cell lines A2780, SKOV3, OVCAR8 and OVCAR3 by western blot. (**B**) Western blot was used to detect the expression pattern of UQCRFS1 in A2780 and OVCAR-8 cells after transfection with siRNA for 24 h (n = 3). (**C**) CCK8 assays were performed to evaluate the proliferation of A2780 and OVCAR8 cells after UQCRFS1 knockdown at 0 h, 24 h, 48 h, 72 h (n = 4). (**D**–**G**)UQCRFS1 knockdown caused G0/G1 phase accumulation, as measured flow cytometry. (t-test for G0/G1 phase and *P < 0.05, **P < 0.01). (**H**–**K**) Western blotting was used to detect the expression of CDK2, CDK4 and CyclinD1 in A2780 and OVCAR8 cells after knocking down UQCRFS1. GAPDH was used as internal control (n = 3). In the bargraphs, the data are shown as the mean ± SD (*P < 0.05, **P < 0.01).

To verify whether the altered expression of UQCRFS1 would affect the growth of ovarian cells, we transfected small interfering RNA (siRNA) to knockdown UQCRFS1. We selected A2780 and OVCAR8 cells with relatively high expression of UQCRFS1 from four ovarian cancer cell lines (Fig. 5A), and western blot analysis showed that UQCRFS1 was stably knocked down (Fig. 5B). Then, the proliferation of the transduced cells was compared with that of control cells to assess the functions of UQCRFS1. When UQCRFS1 was knockdown, cell proliferation significantly slowed (Fig. 5C). These results indicated that high expression of UQCRFS1promoted the proliferation of OC cells.

### Knockdown of UQCRFS1 induces G1 arrest in OC cells

Cell cycle regulation is closely linked to cell proliferation^12^. We tested whether UQCRFS1 knockdown affects cell cycle alterations by flow cytometry. The results showed a significantly increased percentage of G1-phase cells among A2780 and OVCAR8 cells with UQCRFS1 knockdown compared with the corresponding control-transduced cells. In contrast, the percentage of S-phase cells significantly decreased in the knockdown cells (Fig. 5D–G). In addition, the expression of cell cycle regulatory proteins, including cyclin D1, CDK2, and CDK4, was significantly decreased in UQCRFS1-knockdown cells (Fig. 5H–K). These findings indicate that high expression of UQCRFS1promotes proliferation in OC cells by regulating the cell cycle.

### Knockdown of UQCRFS1 induces apoptosis of OC cells

**Figure 6:**
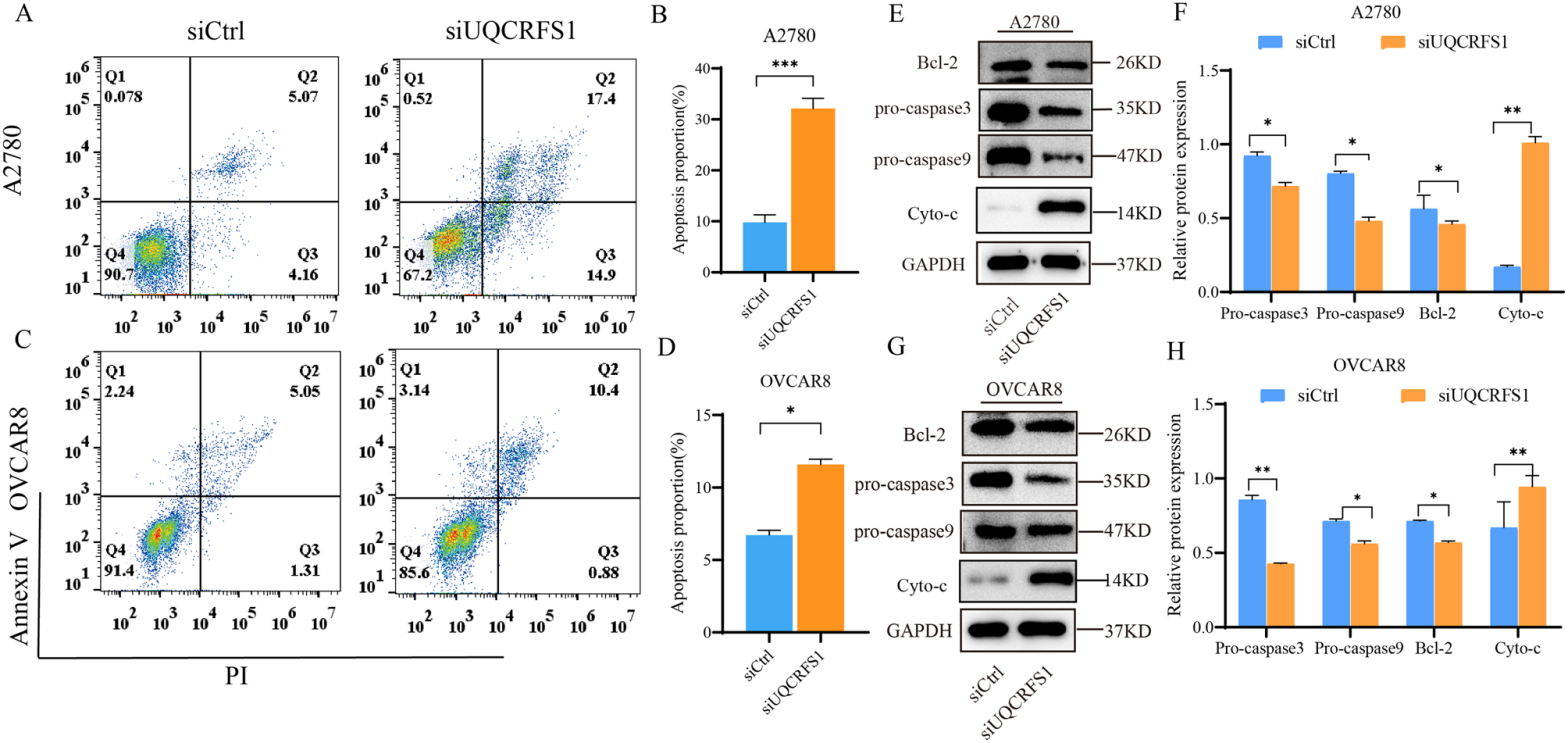
Knockdown of UQCRFS1 induces apoptosis of OC cells. (**A**–**D**) Cells were collected and stained with FITC-conjugated annexin V and PI and subjected to flow cytometry. Knockdown of UQCRFS1 increased the proportion of A2780 and OVCAR8 apoptotic cells (t-test, **P < 0.01 ***P < 0.001). (**E**–**H**) The expressions of pro-caspase3, pro-caspase-9, Cyto-c and Bcl-2 were determined after knocking down UQCRFS1 by western blot analysis. GAPDH was used as internal control (n = 3). In the bargraphs, the data are shown as the mean ± SD (*P < 0.05, **P < 0.01 ***P < 0.001).

To further explore whether the expression of UQCRFS1 affects apoptosis in OC cells, we performed double-staining with PI together with Annexin V and analysed the results by flow cytometry. UQCRFS1-knockdown significantly increased the percentage of apoptotic cells, compared with the control group, in both A2780 and OVCAR8 cells (Fig. 6A–D). Moreover, western blot analysis detected the expression of apoptosis signalling proteins. When UQCRFS1 knockdown, pro-caspase3, 9, Cyto-c, and Bcl-2 protein expression was dramatically reduced (Fig. 6E–H). These results suggest that UQCRFS1 upregulation increases the anti-apoptosis of OC.

### UQCRFS1 knockdown induces ROS production and DNA damage genes expression

**Figure 7:**
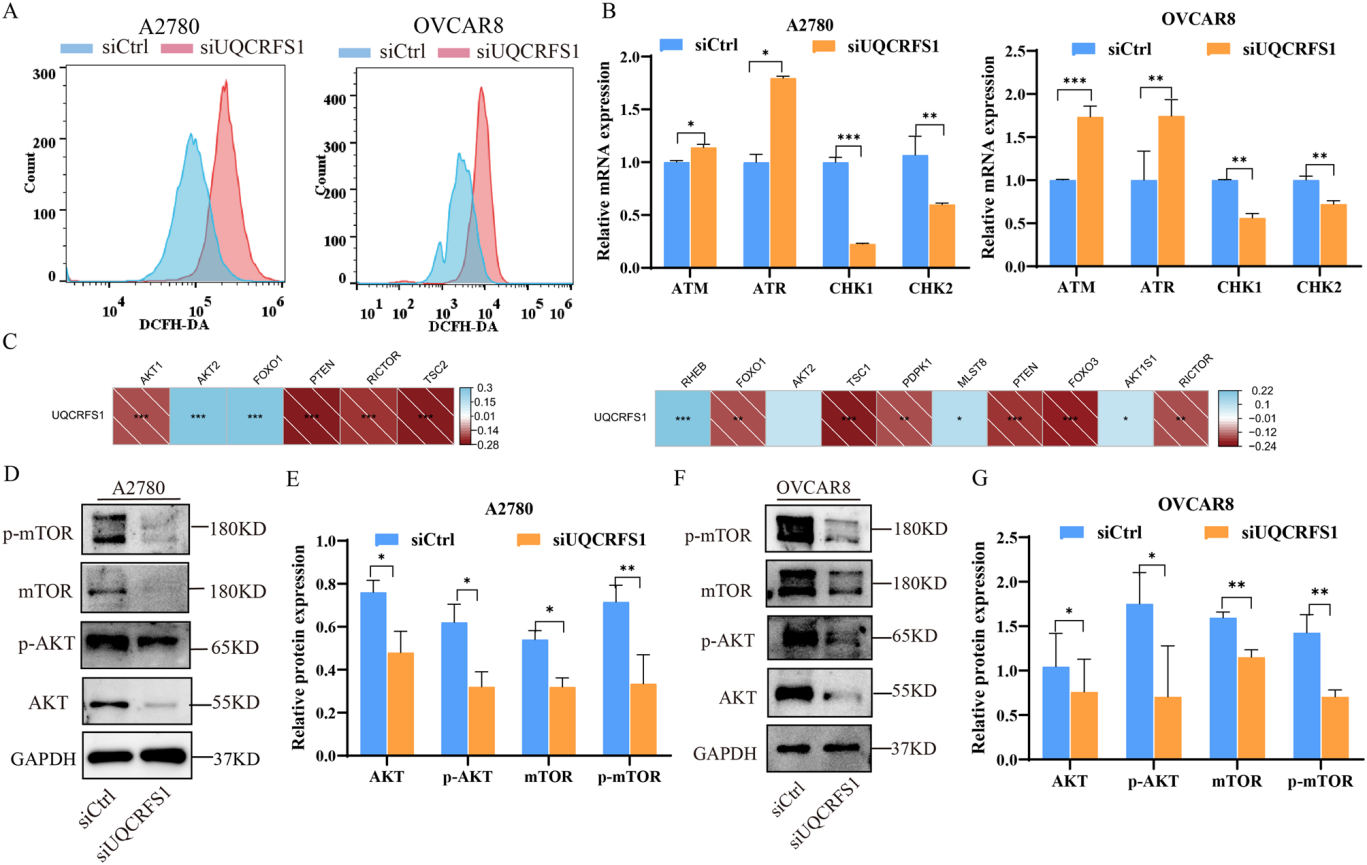
Knockdown of UQCRFS1 triggers changes in the DNA damage genes, ROS production and AKT/mTOR signaling pathway. (**A**) Flow cytometry was used to detect ROS levels in A2780 and OVCAR8 cells after knockdown of UQCRFS1 by loading the DCFH-DA probe, and the peak shifted to the right and ROS production increased (t-test, *P* < 0.05) (**B**) Relative mRNA expression of ATM, ATR, CHK1 and CHK2 in A2780 and OVCAR8 cells after knockdown of UQCRFS1. (**C**) Correlation between AKT/mTOR signature and UQCRFS1. (**D**–**G**) Western blot of AKT, p-AKT, mTOR, p-mTOR in siCtrl and siUQCRFS1 about A2780 and OVCAR8 (n = 3). In the bargraphs, the data are shown as the mean ± SD (*P < 0.05, **P < 0.01 ***P < 0.001).

The mitochondrial respiratory chain is the leading cellular site for ROS production. ROS production is affected when the mitochondrial respiratory chain disturbance, resulting in DNA damage^13^. We investigated the generation of ROS in UQCRFS1 knockdown cells using DCFH-DA and flow cytometry. As shown in Fig. 7A, a markedly enhanced fluorescent signal was observed in UQCRFS1 knockdown cells compared to the signal in control cells. It suggests that UQCRFS1-knockdown increased ROS production. Next, we wanted to determine if excess ROS caused DNA changes. We investigated the mRNA expression of DNA damage genes by RT-qPCR. UQCRFS1-knockdown notably upregulated the mRNA expression of ATM and ATR, whereas CHK1 and CHK2 were down-regulated. (Fig. 7B). These results suggest that UQCRFS1 knockdown results in accumulation of ROS, as well as DNA damage.

### Knockdown of UQCRFS1 triggers changes in the AKT/mTOR signalling pathway

According to previous reports, the Akt/mTOR signalling pathway allows cells to maintain circulation, an essential factor in promoting cell growth and proliferation. Correlation analysis was performed between UQCRFS1 expression and AKT/mTOR pathway datasets to gain further insight into the molecular mechanism involved in OC pathogenesis. The related indicators of the AKT/mTOR pathway, such as PTEN RICROR, were negatively correlated with high expression of the UQCRFS1 gene (Fig. 7C), indicating that UQCRFS1 may be an activator of the AKT/mTOR pathway. We evaluated the protein expression levels of the AKT/mTOR signalling pathway by western blot. As shown in Fig. 7D–G, total AKT, mTOR, p-AKT and p-mTOR expression were reduced in UQCRFS1-knockdown cells. These findings demonstrate that the AKT/mTOR pathway may be involved in cell cycle arrest and apoptosis caused by UQCRFS1 knockdown.

## Discussion

Recent studies have shown that mitochondria are not only energy providers to cells but also mediators of cell signalling, metabolic regulation, cell cycle control, cell development and death^14^. Emerging data demonstrate that abnormalities in the components and function of mitochondria are associated with prognosis and progression in many human cancers. There is evidence that targeting metabolism or mitochondrial proteins may be a new approach for carcinoma treatment^15^. Here, we investigated UQCRFS1, an essential mitochondrial respiratory complex III subunit. It may be a reliable prognostic biomarker and molecular target for treating OC.To investigate the involvement of UQCRFS1 in OC, public online websites, such as GEPIA and HPA, were used to evaluate the expression of this gene in OC patients. Western blotting and IHC results also demonstrated that UQCRFS1 high-expression in OC tumour tissues compared with normal tissues, which has the potential to predict the prognosis of patients. However, the significance of UQCRFS1 and its mechanism of action in the progression of OC have yet to be reported.

We found that UQCRFS1 was highly expressed in ovarian cancer and was associated with poor prognosis. To further explore the function of UQCRFS1, we did a correlation analysis between the UQCRFS1 gene and the tumour-related dataset. We found that the UQCRFS1 gene positively correlated with cell cycle, OXPHOS and negatively correlated with apoptosis and DNA damage. Therefore, UQCRFS1 plays a part in the development of OC.

We knocked down UQCRFS1 and found that cell proliferation was inhibited. The cell cycle plays a crucial role in regulating cell growth. According to our data, the knockdown of UQCRFS1 arrested the cell cycle at G1 and decreased the expression of cyclinD1, CDK2, and CDK4 proteins. These in vitro results may provide an experimental basis for the role of UQCRFS1 in the development of OC. Meanwhile, further in vivo studies are ongoing to verify the efficiency of UQCRFS1-targeted therapy in OC. These findings further support our hypothesis that UQCRFS1 promotes the growth of OC.

In the present study, we noticed that the expression of mitochondrial respiratory chain complex genes was elevated in the UQCRFS1 high-expression group, providing the possibility for enhanced mitochondrial respiration. It suggests that high UQCRFS1 expression is critical for maintaining mitochondrial function. Some studies have shown that, in contrast to Warburg’s effect, mitochondrial respiratory chain function is vital in some tumours, such as breast cancer^16^, melanoma^17^, and OC^18^. In agreement with the results of previous studies, we detected UQCRFS1 overexpression in 35 OC samples. This phenomenon suggests that mitochondrial respiration is activated to provide energy in most OC patients, providing clinical evidence for the previous theory. It is worth pointing out that our conclusions do not rule out the glycolytic pathway in OC. There is evidence that glycolysis is essential for the development of OC^19^. Therefore, how both oxidative phosphorylation and glycolysis are tumorigenic in OC remains to be further explored. However, at least, we show that UQCRFS1 is highly expressed in OC, thus providing more energy for cancer development, which may make UQCRFS1 a potential target for OC therapy.

Mitochondrial dysfunction causes ROS production accumulation and abnormal redox homeostasis. Excessive accumulation of ROS leads to increased oxidative stress, DNA damage, and genomic instability^20^. DNA damage induces cell cycle arrest in eukaryotic cells through checkpoint activation or DNA repair mechanisms to repair damaged DNA. However, damaged cells undergo apoptosis when DNA damage cannot be repaired. The DNA damage response has primarily been defined in the context of the ATM-CHK2 pathway and the ATR-CHK1 pathway^21^. However, this study showed that ATM and ATR were activated, and CHK1 and CHK2 were inactivated, which is inconsistent with previous reports. Previous studies have shown that ATM-CHK2 and ATR-CHK1 signal transduction also require multiple kinases. When ATR is activated, it is required to activate downstream substrate proteins, such as claspin-mediated proteins, which activate CHK1 and further cause cell cycle arrest. Mutations in these phosphorylation sites of Claspin inhibit the Claspin-CHK1 interaction in vivo and impair CHK1 activation, leading to replication checkpoint defects^22^. Therefore, we explain that the failure of CHK1 activation may be due to a change in the intermediate kinase.

Moreover, in p53-intact cells, checkpoint function is mainly mediated by CHK1 and p53 responses downstream of ATM. However, CHK1 is dispensable in the presence of a functional ATM/p53/p21 pathway. The role of CHK1 is redundant if other checkpoint regulators are active, or it may interfere with other pathways that control cell proliferation, such as pRb phosphorylation^23^. However, in p53-deficient cancer cells, checkpoint signaling after DNA damage is mediated through the combined action of CHK1 and p38 MAPK/MK2 pathways. The p38MAPK/MK2 branch is essential for cell survival after DNA damage^24^. Using CHK1 inhibitors also results in replication fork arrest and DNA fragmentation, which leads to apoptosis^25^. That means ATM-CHK1 is not the only pathway responsible for cell cycle arrest after DNA damage. In this study, we found that when ROS is overproduced, it leads to DNA damage, which possibly activates pathways other than ATM-CHK1 for cell cycle arrest. Meanwhile, we detected that the proportion of apoptosis increased after knocking down UQCRFS1.The unsuccessful activation of CHK1 also exacerbates apoptosis. The specific molecular mechanism involved is also the direction we would explore further.

According to Lokendra Kumar Sharma et al. research, mitochondrial complex I abnormalities lead to tumour formation by causing oxidative stress and activating the AKT pathway^26^. Qing Wang et al.^27^ used RNA-Seq to find substantial alterations in the AKT pathway when knockout UQCRC1 in pancreatic cancer investigation, verified by Western blot. Bojie Chen et al.^28^ discovered that reversible mitochondrial inhibition might limit tumour growth, migration, and invasion via the PI3K/Akt/FoxO1/Cyclin D1 pathway for papillary thyroid cancer. Moreover, Mingming Sun et al. discovered that PIKE-A (phosphoinositol 3-kinase enhancer activated Akt) influences the expression of respiratory chain complex II succinate dehydrogenase A (SDHA) via the STAT3/FTO axis, hence influencing mitochondrial activity^29^. In work by Jiao-Yan Cheng et al., AKT inhibitors substantially affected the location of PHB1 in mitochondria, which is essential for mitochondrial function^30^. Overall, there is an interaction between AKT and mitochondria. Therefore, we hypothesized that the AKT pathway would also alter if mitochondrial complex III altered in ovarian cancer. After the knockdown of UQCRFS1, the expression of total AKT, phosphor-AKT, total mTOR, and phosphor-mTOR was reduced. It indicates that the Akt/mTOR pathway may be involved in UQCRFS1 knockdown-induced changes in biology. Of course, the particular mechanism must still be further investigated.

## Conclusion

In conclusion, we found that UQCRFS1 is more highly expressed in OC and that the expression level correlates with the prognosis of OC. Furthermore, our results indicate that UQCRFS1 affects tumour cell proliferation, cell cycle, apoptosis, and DNA damage, which may be related to the AKT/mTOR signalling pathway. These results prove that UQCRFS1 is a prognostic biomarker for OC and a potential therapeutic target. However, further explorations are needed to elucidate its role in OC progression in vivo fully.

## Supplementary Information

Supplementary Information 1.

Supplementary Tables.

Supplementary Information 2.

## Data Availability

The datasets analyzed during the current study are available in the TCGA database (https://tcgadata.nci.nih.gov/tcga/), GTEx database (https://commonfund.nih.gov/GTEx), GEO database (https://www.ncbi.nlm.nih.gov), GEPIA database (http://gepia2.cancer-pku.cn/), GEPIA database (https://www.proteinatlas.org/), the c-BioPortal database (http://www.cbioportal.org/).
