# Supplementary figures and images for: UQCRFS1 serves as a prognostic biomarker and promotes the progression of ovarian cancer
Source: Sci Rep. 2023 May 23;13:8335. doi: 10.1038/s41598-023-35572-z (PMC10205806; doi:10.1038/s41598-023-35572-z)

Clinical data supplement of UQCRFS1.


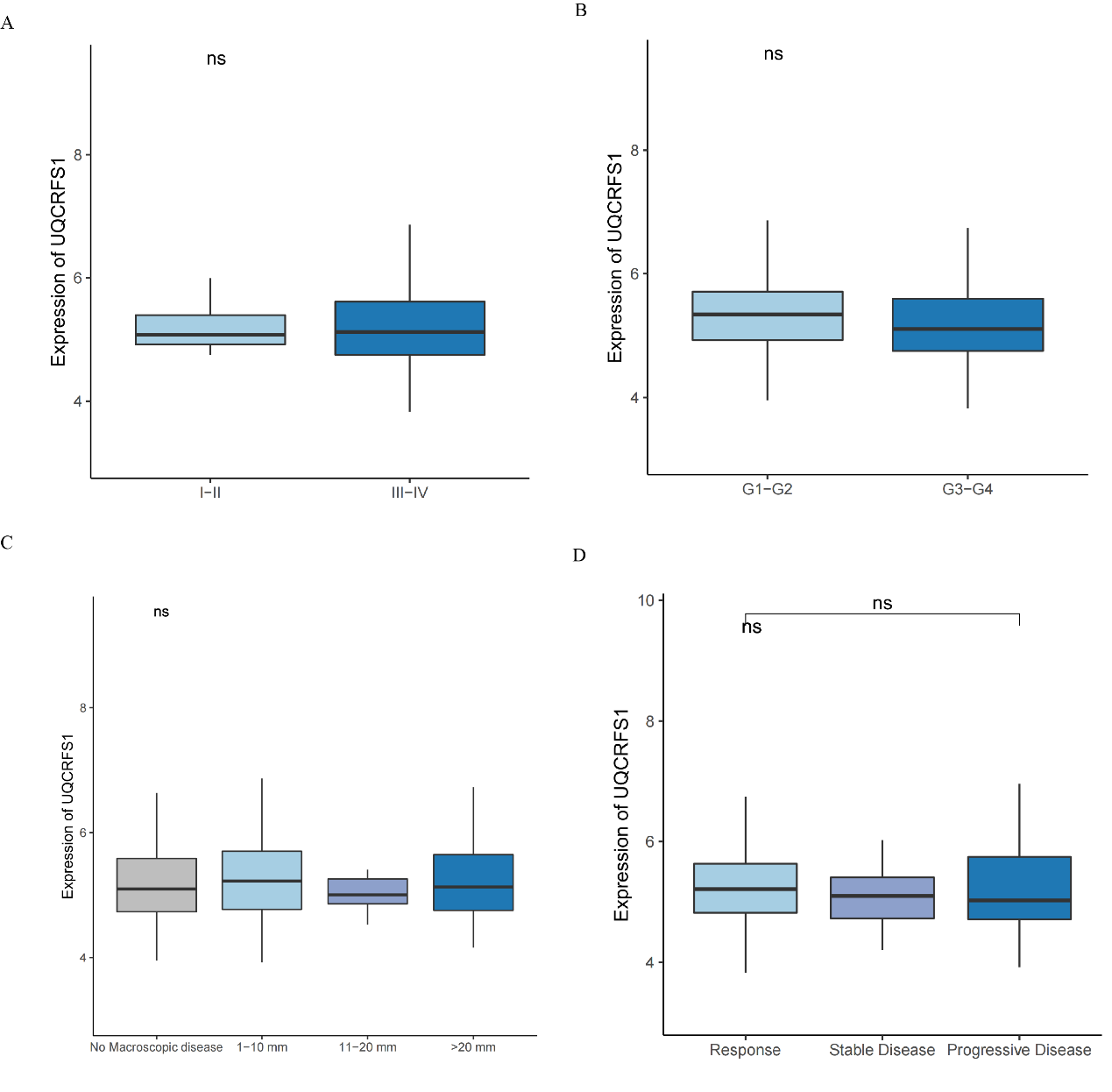

Supplement: Supplementary file 1 — Supplementary Information 1. [file 41598_2023_35572_MOESM1_ESM.docx]
