# Supplementary material for: UQCRFS1 serves as a prognostic biomarker and promotes the progression of ovarian cancer
Source: Sci Rep. 2023 May 23;13:8335. doi: 10.1038/s41598-023-35572-z (PMC10205806; doi:10.1038/s41598-023-35572-z)
Supplement: Supplementary file 2 — Supplementary Tables. [file 41598_2023_35572_MOESM2_ESM.docx]

**Table. S1 Primer sequences**

| GENE | Forward primer | Reverse primer |
| --- | --- | --- |
| CHK1 | ATATGAAGCGTGCCGTAGACT | TGCCTATGTCTGGCTCTATTCTG |
| CHK2 | GCTGGGTATAACCGTGCTGT | CGTAAAACGTGCCTTTGGAT |
| ATM | TTGATCTTGTGCCTTGGCTAC | TATGGTGTACGTTCCCCATGT |
| ATR | TCCCTTGAATACAGTGGCCTA | TCCTTGAAAGTACGGCAGTTC |
| GAPDH | GAGCGATGGTAGTTCTGGA | CCCGATGCTCTTCACCT |

**Table.S2. univariate Cox model for overall survival and progression-free survival**

|  | PFS | | | |  | OS | | | |
| --- | --- | --- | --- | --- | --- | --- | --- | --- | --- |
| id | HR | HR.95L | HR.95H | P |  | HR | HR.95L | HR.95H | P |
| C19orf33 | 1.654 | 1.225 | 2.232 | 0.001 |  | 1.438 | 1.047 | 1.976 | 0.025 |
| UQCRFS1 | 1.506 | 1.136 | 1.997 | 0.004 |  | 1.644 | 1.215 | 2.223 | 0.001 |
| VGLL1 | 1.521 | 1.139 | 2.030 | 0.004 |  | 1.385 | 1.017 | 1.884 | 0.038 |
| KLHL14 | 1.443 | 1.118 | 1.862 | 0.005 |  | 1.529 | 1.162 | 2.013 | 0.002 |
| RHPN2 | 1.376 | 1.065 | 1.778 | 0.015 |  | 1.485 | 1.129 | 1.953 | 0.005 |
| CHMP4C | 1.304 | 1.001 | 1.698 | 0.049 |  | 1.364 | 1.02 | 1.823 | 0.036 |

**Table.S3 multivariate Cox analysis**

| id | HR | HR.95L | HR.95H | P |
| --- | --- | --- | --- | --- |
| C19orf33.3 | 1.374 | 0.998 | 1.892 | 0.051 |
| grade | 1.015 | 0.672 | 1.532 | 0.943 |
| age | 1.02 | 1.006 | 1.033 | 0.003 |
| stageIV | 1.23 | 0.86 | 1.759 | 0.258 |
| KLHL14.3 | 1.552 | 1.17 | 2.059 | 0.002 |
| grade | 1.139 | 0.748 | 1.734 | 0.544 |
| age | 1.02 | 1.007 | 1.033 | 0.003 |
| stageIV | 1.305 | 0.911 | 1.869 | 0.146 |
| CHMP4C.3 | 1.32 | 0.983 | 1.774 | 0.065 |
| grade | 1.004 | 0.665 | 1.514 | 0.986 |
| age | 1.019 | 1.006 | 1.032 | 0.004 |
| stageIV | 1.294 | 0.903 | 1.854 | 0.161 |
| VGLL1.3 | 1.351 | 0.991 | 1.842 | 0.057 |
| grade | 0.975 | 0.646 | 1.473 | 0.905 |
| age | 1.02 | 1.007 | 1.034 | 0.002 |
| stageIV | 1.204 | 0.84 | 1.725 | 0.312 |
| RHPN2.3 | 1.521 | 1.151 | 2.01 | 0.003 |
| grade | 0.968 | 0.644 | 1.456 | 0.876 |
| age | 1.022 | 1.009 | 1.036 | 0.001 |
| stageIV | 1.153 | 0.804 | 1.655 | 0.439 |
| UQCRFS1.3 | 1.607 | 1.186 | 2.178 | 0.002 |
| grade | 1.04 | 0.688 | 1.571 | 0.853 |
| age | 1.02 | 1.006 | 1.033 | 0.003 |
| stageIV | 1.219 | 0.853 | 1.744 | 0.277 |
