# Supplementary material for: UQCRFS1 serves as a prognostic biomarker and promotes the progression of ovarian cancer
Source: Sci Rep. 2023 May 23;13:8335. doi: 10.1038/s41598-023-35572-z (PMC10205806; doi:10.1038/s41598-023-35572-z)
Supplement: Supplementary file 3 — Supplementary Information 2. [file 41598_2023_35572_MOESM3_ESM.docx]

Fig 2 western blot original image


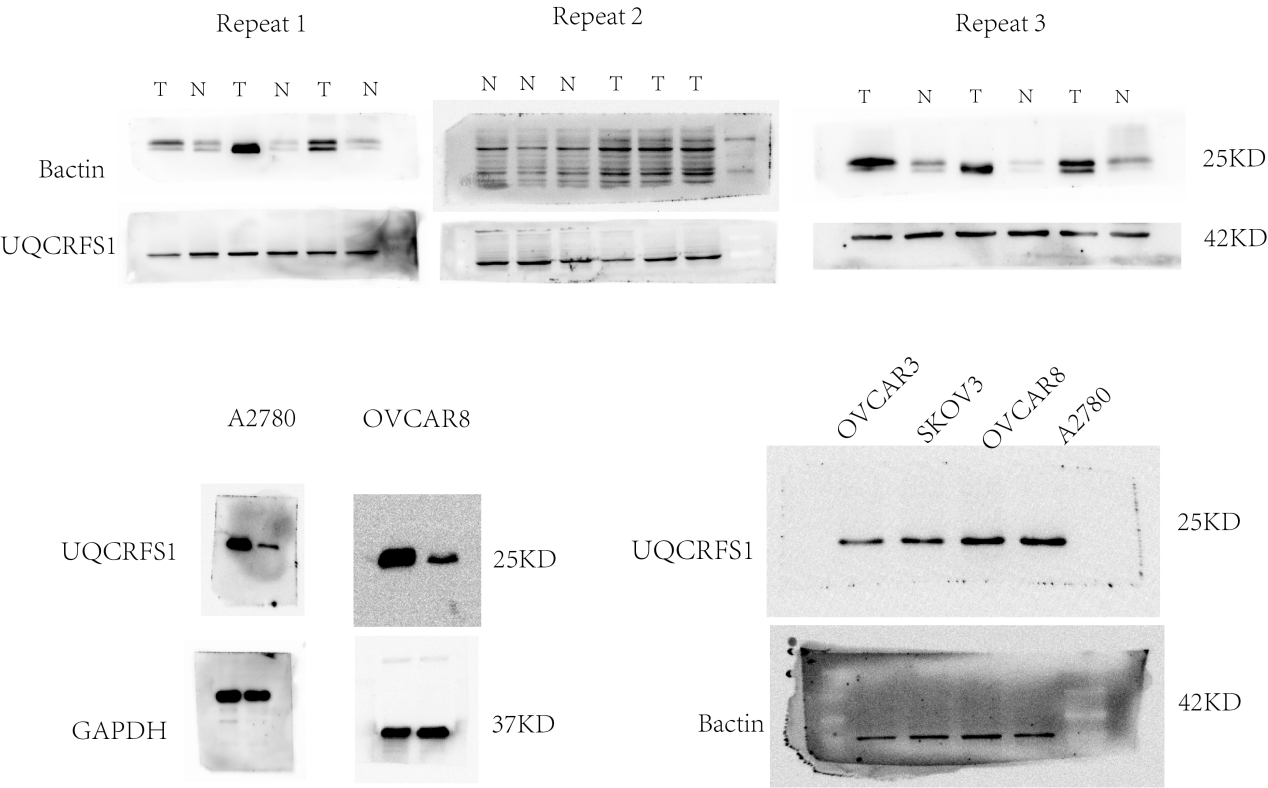


Fig 5 western blot original image


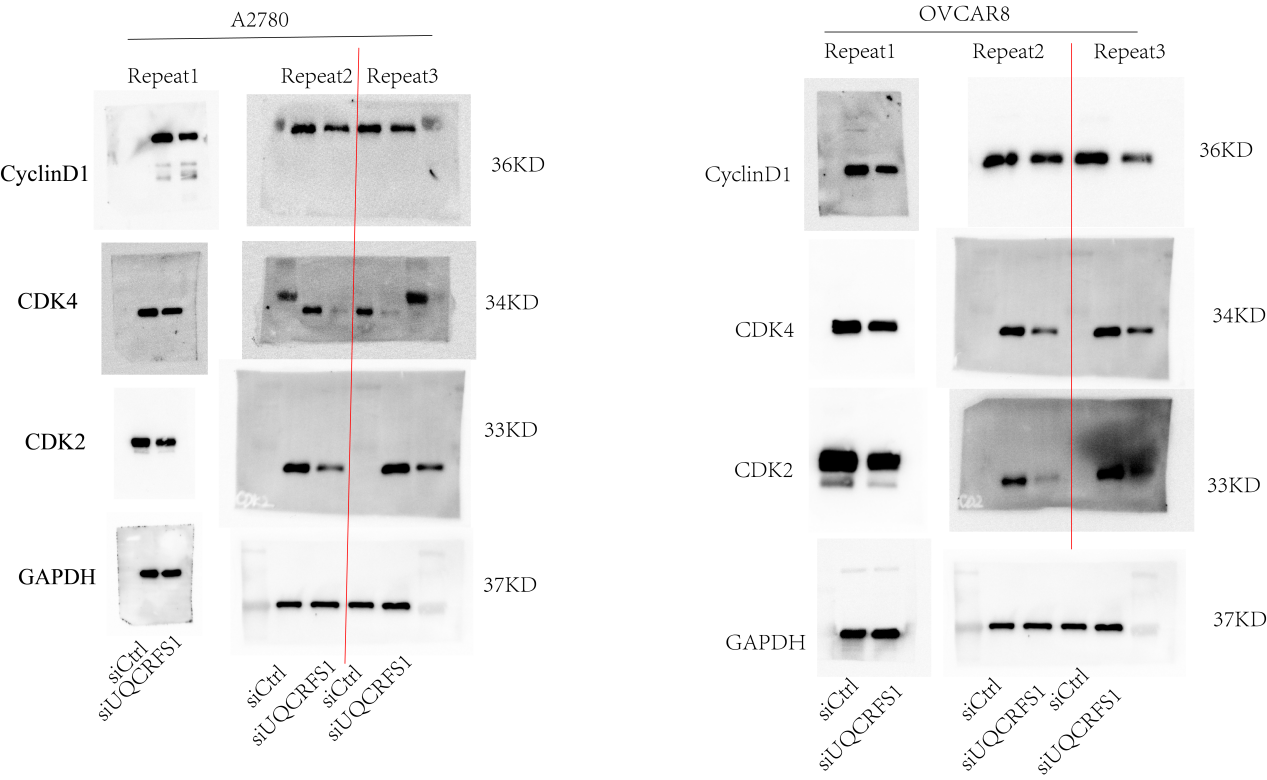


Fig 6 western blot original image


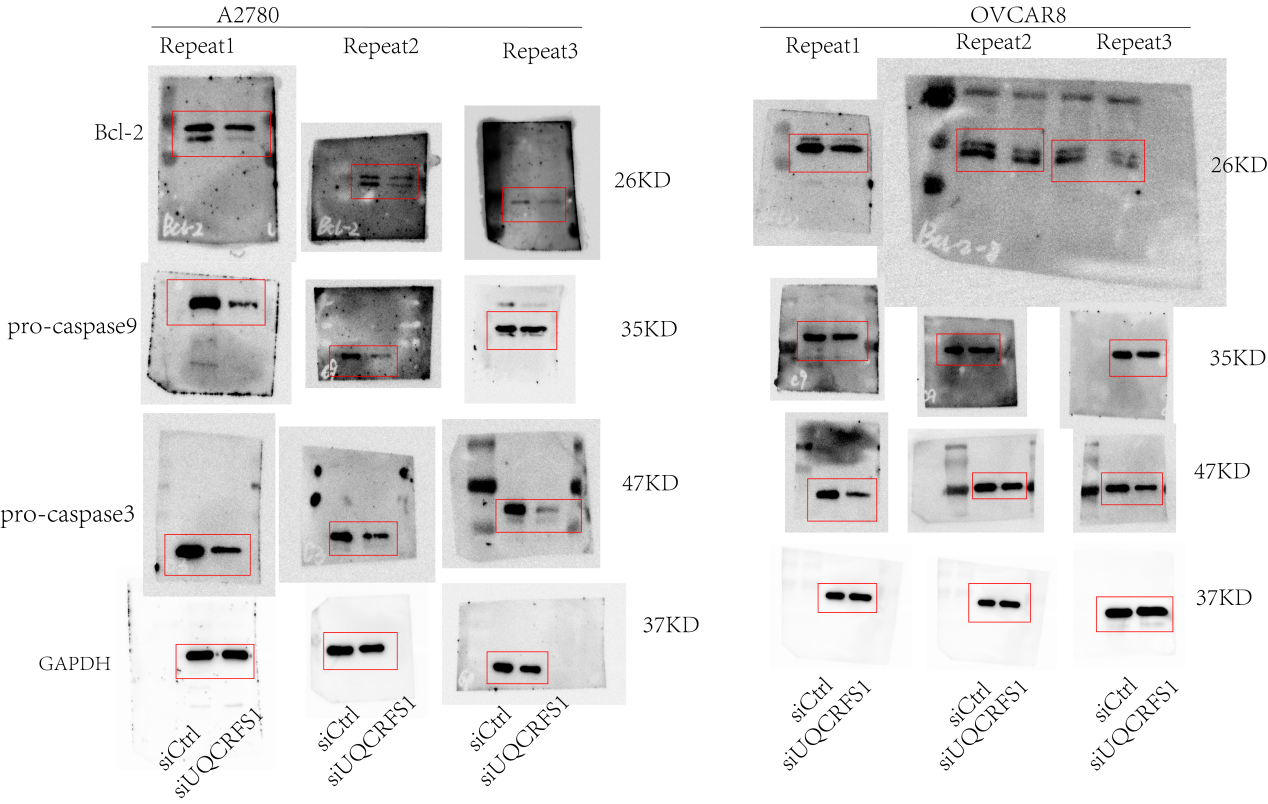


Cyto-c western blot original image


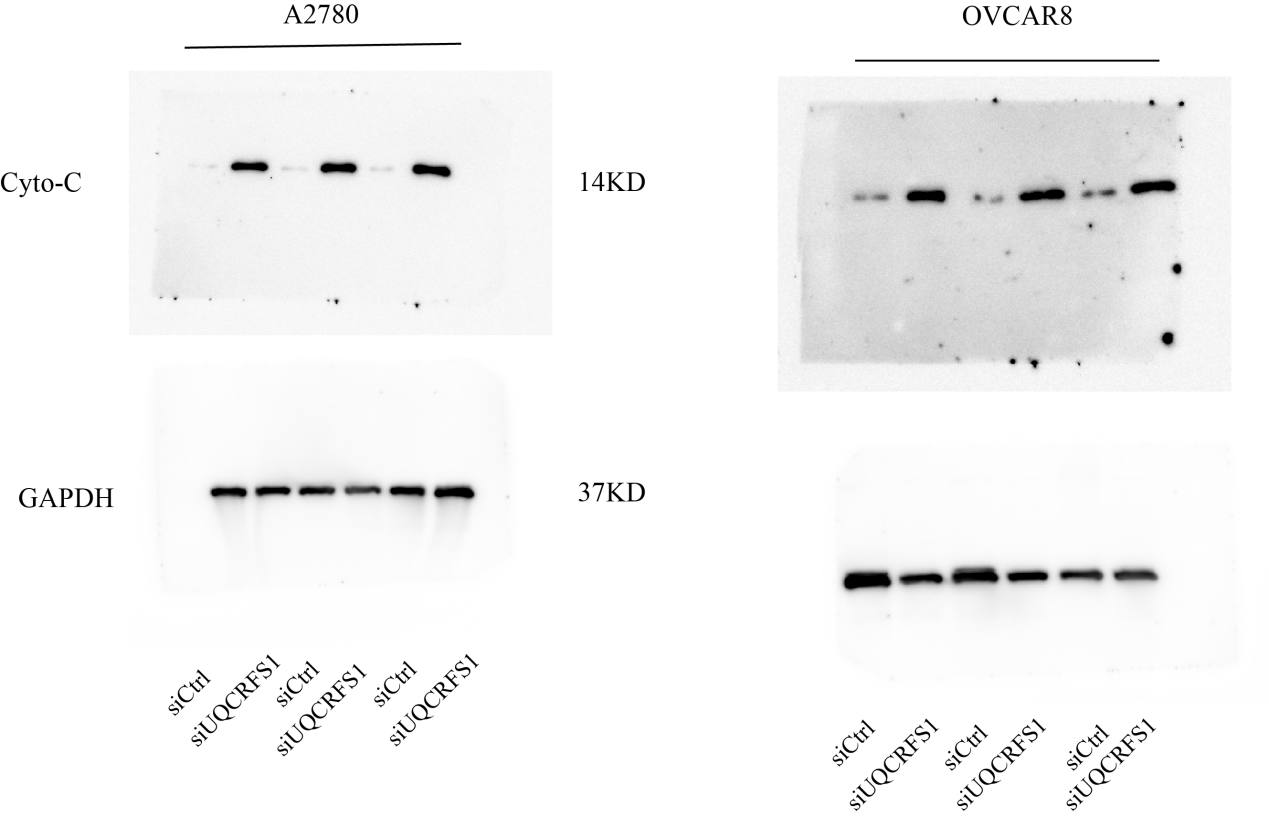


Fig 7 western blot original image


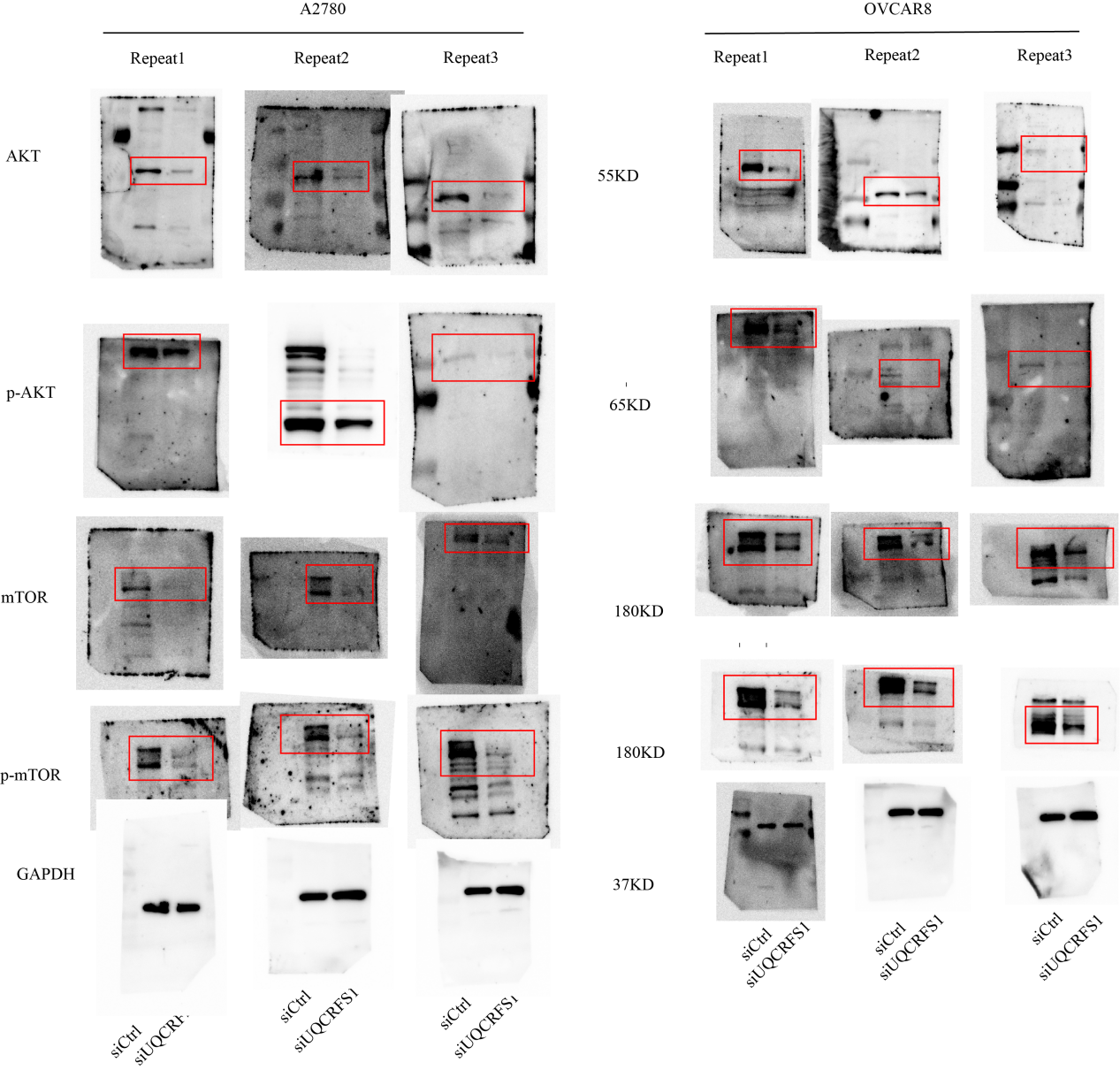
­­­
